# IL‐7–Mediated IL‐7R‐JAK3/STAT5 signalling pathway contributes to chemotherapeutic sensitivity in non–small‐cell lung cancer

**DOI:** 10.1111/cpr.12699

**Published:** 2019-10-10

**Authors:** Lin Shi, Zhaozhong Xu, Qiong Yang, Yuanyuan Huang, Yuxin Gong, Fang Wang, Bin Ke

**Affiliations:** ^1^ Department of Traditional Chinese Medicine Zhujiang Hospital of Southern Medical University Guangzhou China; ^2^ Department of Emergency Zhujiang Hospital of Southern Medical University Guangzhou China; ^3^ Department of Oncology Sun Yat‐sen Memorial Hospital of Sun Yat‐sen University Guangzhou China; ^4^ Department of VIP Ward Affiliated Cancer Hospital of Sun Yat‐Sen University Guangzhou China; ^5^ Department of Respiratory Diseases Zhujiang Hospital of Southern Medical University Guangzhou China; ^6^ Department of Oncology The First Affiliated Hospital of Sun Yat‐sen University Guangzhou China; ^7^ Department of Traditional Chinese Medicine The First Affiliated Hospital of Sun Yat‐sen University Guangzhou China

**Keywords:** cisplatin resistance, IL‐7, IL‐7R, JAK3, NSCLC, STAT5

## Abstract

**Objectives:**

The chemotherapy drug resistance is a major challenge for non‐small‐cell lung cancer (NSCLC) treatment. Combination of immunotherapy and chemotherapy has shown promise for cancer. The goal of this study was to evaluate the anti‐tumour efficacy of interleukin‐7 (IL‐7) combining cisplatin against NSCLC.

**Materials and Methods:**

Cell proliferation was analysed using CCK‐8 assay, EdU proliferation assay and colony‐forming assay. Cell apoptosis was evaluated using HOECHST 33342 assay and flow cytometry. The protein expression levels were analysed by Western blot. The blocking antibody against the IL‐7 receptor and the inhibitors of STAT5 and JAK3 were used to investigate the pathway involved. A xenograft model was established to assess the anti‐tumour efficacy of IL‐7 combining cisplatin in vivo.

**Results:**

Here we found IL‐7R was increased in A549/DDP cells compared with A549 cells. The block of IL‐7R reversed the inhibitory effects of IL‐7 combined with cisplatin and decreased the numbers of apoptosis cells induced by treatment of IL‐7 combined with cisplatin. The JAK3 inhibitor and STAT5 inhibitor were used to identify the pathway involved. The results showed that JAK3/STAT5 pathway was involved in enhancing role of cisplatin sensitivity of NSCLC cells by IL‐7. In vivo, cisplatin significantly inhibited tumour growth and IL‐7 combined with cisplatin achieved the best therapeutic effect.

**Conclusion:**

Together, IL‐7 promoted the sensitivity of NSCLC cells to cisplatin via IL‐7R‐JAK3/STAT5 signalling pathway.

## 1 INTRODUCTION

As one of the most common cancer types in women and men, lung cancer is the leading cause of cancer‐related morbidity and mortality worldwide, representing 13% of newly diagnosed new cancer cases.^1^, ^2^ NSCLC accounts for about 85% of all lung cancer cases and has a high incidence of cancer recurrence and metastasis, which leads to the failure in treatment of NSCLC.^3^, ^4^ Although early diagnosis and therapeutic approaches of NSCLC patients have considerably progressed, the total 5‐year survival rate of NSCLC patients is less than 15%.^5^ Currently, radiotherapy and chemotherapy for NSCLC are the standard treatment options and have a certain efficacy. However, the toxicity and side effects have limited its implementation in these patients.^6^, ^7^ Importantly, the chemotherapy drug resistance limits the clinical efficacy of therapies for NSCLC, which leads to the main recurrence and metastasis of NSCLC.^4^, ^8^ The development of novel strategies for lung cancer is still critical.

Platinum‐based chemotherapy regimens, particularly cisplatin, are a standard adjuvant therapeutic strategy in advanced stage NSCLC.^9^, ^10^ Nowadays, the incidence of cisplatin resistance of NSCLC is up to 63%. In cells, cisplatin binds to DNA in nuclei and mitochondria to form the cisplatin‐DNA adducts, which destroys DNA by blocking DNA replication and transcription and induces cell cycle arrest and apoptosis.^11^, ^12^ The NSCLC cells develop the chemoresistance to cisplatin by increasing drug detoxification, changes in DNA repair, DNA damage response, DNA damage tolerance, cell cycle checkpoints and reduced cell apoptosis.^13^, ^14^, ^15^, ^16^, ^17^, ^18^ Therefore, identifying novel molecular‐targeted therapeutic approaches to overcome cisplatin resistance or developing safe methods to reverse drug resistance is essential for the treatment of NSCLC.

Growing evidence has demonstrated that combination of immunotherapy and chemotherapy has shown promise in the treatment of NSCLC. Interleukin‐7 (IL‐7) plays an important role in affecting T‐cell proliferation, development and homeostasis.^19^, ^20^, ^21^, ^22^ Previous studies have reported that administration of IL‐7 combined with oxaliplatin significantly suppressed the growth of tumours in lung and abdomen metastasis models of colon cancer.^23^ For NSCLC, whether the IL‐7 combining cisplatin has a better anti‐tumour activity and reverses drug resistance, however, is still unclear.

Here, we have investigated whether IL‐7 affects the chemotherapeutic sensitivity of NSCLC cells to cisplatin, and showed that IL‐7 enhanced the sensitivity of NSCLC cells. We have also showed that IL‐7 enhanced the sensitivity of A549/DDP cells to cisplatin. To investigate the roles of IL‐7/IL‐7R signalling pathway in enhancing the sensitivity of A549 cells to cisplatin, a blocking antibody against the IL‐7 receptor was used. We found that the block of IL‐7R reversed the inhibitory effects of IL‐7 combined with cisplatin and decreased the numbers of apoptosis cells induced by treatment of IL‐7 combined with cisplatin. The JAK3 and STAT5 inhibitors were used to validate the involvement of JAK3/STAT5 pathway in enhancing the role of cisplatin sensitivity of NSCLC cells by IL‐7. In addition, a xenograft model was established to confirm the anti‐tumour effect of combining IL‐7 and cisplatin in vivo.

## 2 MATERIALS AND METHODS

### 2.1 Cell culture and treatment

NSCLC A549 cell lines were obtained from the Cell Bank of the Chinese Academy of Sciences (Shanghai, China) and cultured in F‐12K medium (GIBCO, USA) supplemented with 10% foetal bovine serum (Gibco, USA), 100 U/mL penicillin and streptomycin (Amresco, Solon, OH) at 37°C in a 5% CO_2_ humidified incubator. The cisplatin‐resistant A549 cell line (A549/DDP) from A549 cells was incubated with gradually increasing cisplatin concentration and maintained in 10% F‐12K medium supplemented with 1 μg/mL DDP. The relative cisplatin resistance was determined by clonogenic assay.

### 2.2 CCK8 assay

The cell viability of cells after indicated treatment was measured by the CCK‐8 assay using the Cell Counting Kit (Dojindo, Japan) according to the manufacturer's instructions. In Brief, 5 × 10^3^ cells/well were seeded into 96‐well plates. A549 cells were treated with 50 ng/mL IL‐7, 1 μg/mL DDP or 50 ng/mL IL‐7 and 1 μg/mL DDP. A549/DDP cells were treated with 5 μg/mL DDP or 50 ng/mL IL‐7 and 5 μg/mL DDP. After the indicated treatment, 10 μL of CCK‐8 reagent was added and incubated with the cells for 2 hours at 37°C. The absorbance of the converted dye at 450 nm was measured using a microplate reader (Thermo Fisher, Finland).

### 2.3 Colony‐forming assay

A total of 1000 A549 cells or A549/DDP cells per well were seeded into 12‐well plate and cultured for 12 days. Then, the colonies were fixed with 20% methanol and stained with 0.1% crystal violet dye. The representative images were photographed, and the colonies were scored.

### 2.4 Quantitative real‐time polymerase chain reaction (qRT‐PCR)

Trizol reagent (Invitrogen) was used to extract total RNA from cells according to the manufacturer's instructions. Reverse transcription reagent kit (Takara, Tokyo, Japan) was used to reverse transcribed into complementary DNA (cDNA). One microgram of total RNA was reverse transcribed to cDNA using a cDNA Reverse Transcription Kit (Takara, Japan). Afterwards, real‐time quantitative PCR (qRT‐PCR) was performed using the SYBR Green PCR Master Mix (Invitrogen, USA) and Step‐One Plus Real‐Time PCR System (Applied Biosystems, CA). The 2^−∆∆CT^ method was used to evaluate the relative expression. The primers used are as follows: IL‐7R forward, 5′‐CCCTCGTGGAGGTAAAGTGC‐3′; reverse, 5′‐CCTTCCCGATAGACGACACTC‐3′; GAPDH forward, 5′‐ TGTGGGCATCAATGGATTTGG‐3′; reverse, 5′‐ ACACCATGTATTCCGGGTCAAT‐3′.

### 2.5 Cell apoptosis assay

After the treatment, the cells were collected, washed, fixed and permeabilized. Then, the cells were stained by Annexin V‐fluorescein isothiocyanate/propidium iodide (Annexin V‐FITC/PI) (BD, CA, USA) according to the manufacturer's instructions. After 15 minutes, the cell apoptosis was measured using flow cytometry (BD Biosciences).

### 2.6 Hoechst 33342 staining

The apoptosis of cells was evaluated using a Hoechst 33342 kit (Thermo Fisher Scientific, MA, USA) according to manufacturer's instructions. In brief, following the treatment for 48 hours, the cells were stained with Hoechst 33342 (10 mg/mL) at 37°C for 10 minutes. Five visual fields were randomly selected from each slide, and approximately 200 cells were counted per field. The FV10i confocal microscope (OLYMPUS, Japan) was used to capture the images.

### 2.7 The xenograft model

To investigate whether IL‐7 enhanced the anti‐tumour efficacy of cisplatin in vivo, a xenograft model was established using the A549 and A549/DDP cells. 5 × 10^6^ A549 or A549/DDP cells resuspended in 200 μL of ice‐cold PBS were injected in each NOD/SCID mice (5 weeks old, male) to establish the xenografts. The mice used in the study were purchased from the Model Animal Research Center of Nanjing University, China. In A549 mouse tumour model, the mice were received the following treatments: DMSO (5%); IL‐7 (5 μg/day); and cisplatin (5 mg/kg) and IL‐7 (5 μg/day) combined with cisplatin (5 mg/kg). In A549/DDP mouse tumour model, the mice were received the following treatments: cisplatin (5 mg/kg); DMSO (5%) combined with cisplatin (5 mg/kg); and IL‐7 (5 μg/day) combined with cisplatin (5 mg/kg). The mice were euthanized on day 28, and the tumour volume was calculated by the following modified ellipsoid formula: (L × W × W)/2, where L is the longitudinal diameter and W is the latitudinal diameter. The animals were housed in groups (N = 6) in 80% humidity with 12‐hour light/dark cycle condition. The mice were euthanized with 10% chloral hydrate after four weeks, and the ectopic tumours were collected. All the animal experiments were approved by the Ethics Committee of the Zhujiang Hospital of Southern Medical University, China.

### 2.8 Immunohistochemical assay

Immunohistochemical assay was performed to analyse the expression of IL‐7R and Ki‐67 as previously described.^24^ The tumour tissue was cut into five μm thick sections and deparaffinized with xylene. IL‐7R (1:100 dilution; Santa Cruz Biotechnology, USA) and Ki‐67(1:500 dilution; Cell Signaling Technology, MA, USA) antibodies were used in the study. Tissue sections were stained with biotinylated secondary antibody (Vector Laboratories, Burlingame, CA, USA). After IHC staining, the sections were counterstained with haematoxylin.

### 2.9 Terminal deoxynucleotidyl transferase dUTP nick‐end labelling (TUNEL) assay

The apoptotic cells were determined using an Apoptag^®^ Peroxidase In Situ Apoptosis Detection Kit (EMD Millipore, Billerica, MA, USA) according to the manufacturer's instructions. Ten fields were randomly selected for the quantification of apoptotic cells at ×20 magnification, and the average counts of TUNEL‐positive cells were calculated.

### 2.10 Western blot assay

Western blot assay was used to detect the protein level in the cells.^2^ BCA Protein Assay Kit (Pierce, USA) was used to analyse the protein concentrations. The JAK3 (1:1500), p‐JAK3 (1:1000), STAT5 (1:1000), p‐STAT5 (1:1000), caspase‐3(1:1000), Bcl‐2(1:1000), Bax (1:1000), IL‐7R (1:1000) and GAPDH (1:2000) antibodies were used in the study. JAK3, p‐JAK3, STAT5, p‐STAT5, caspase‐3, Bcl‐2, Bax and GAPDH antibodies were purchased from Cell Signaling Technology (MA, USA). IL‐7R antibody (1:1500) was purchased from Santa Cruz (USA). The enhanced chemiluminescence reaction was used to detect the protein bands.

### 2.11 Statistical analyses

All results were represented as the mean ± SD. Statistical significance between the groups was analysed by unpaired *t* test, and the differences between more than two groups were analysed by one‐way ANOVA or Kruskal‐Wallis test. *P* value of <.05 was considered statistically significant. Each experiment was performed in triplicates.

## 3 RESULTS

### 3.1 IL‐7 enhanced the sensitivity of NSCLC cells to cisplatin

**Figure 1:**
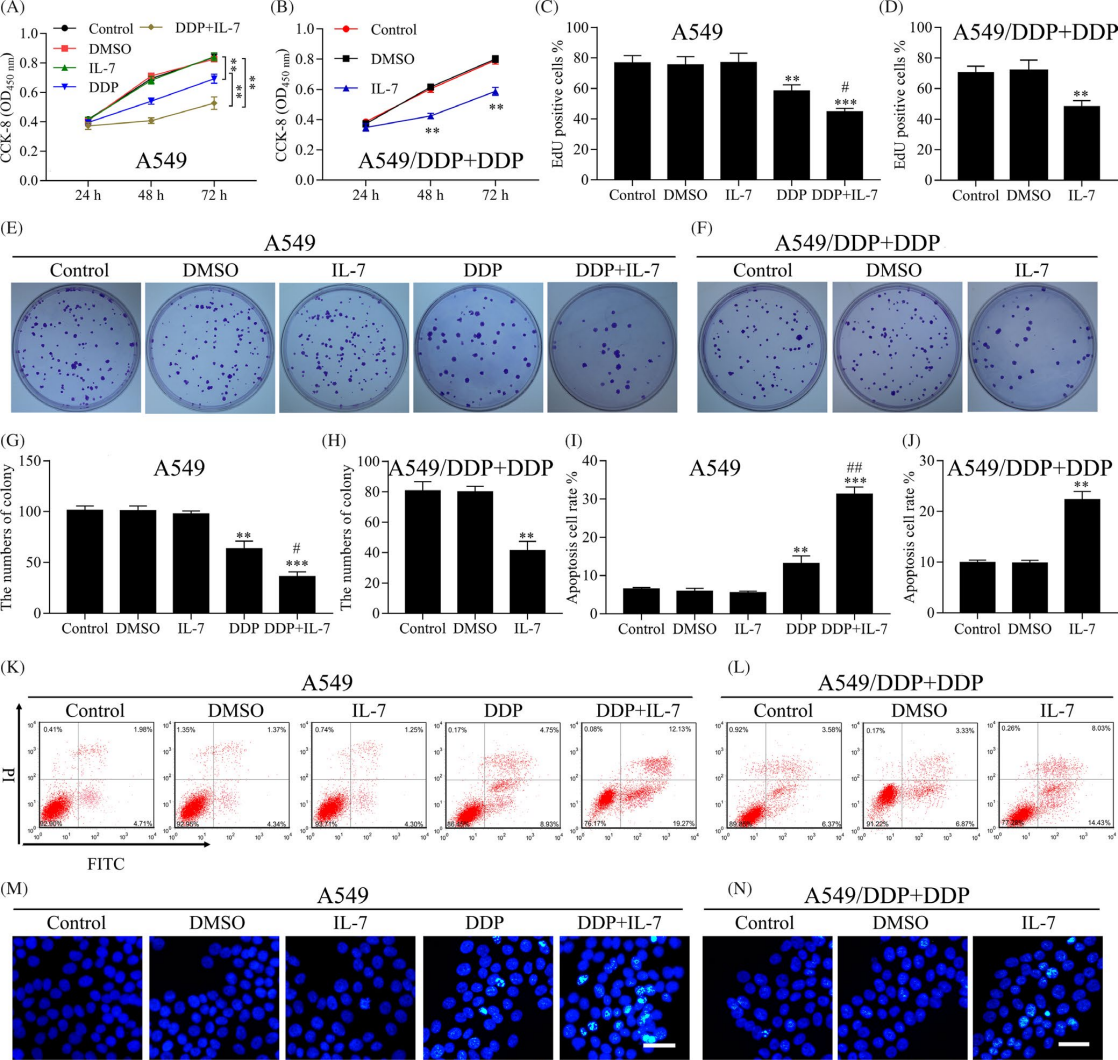
IL‐7 enhanced the sensitivity of NSCLC cells to cisplatin. A, B, Cell proliferation analysis using CCK‐8 assay was performed to assess the cell viability of A549 and A549/DDP cells after indicated treatment. C, EdU proliferation assays were performed on A549 cells after indicated treatment for 48 h, and the percentage of EdU‐positive cells was quantified. DDP group vs DMSO group (**P < .01), IL‐7 group vs DDP + IL‐7 group (***P < .001), DDP group vs DDP + IL‐7 group (#*P* < .05). D, EdU proliferation assays were performed for A549/DDP cells after indicated treatment for 48 h, and the percentage of EdU‐positive cells was quantified. IL‐7 group vs DMSO group (**P < .01). E, F, Colony‐forming assay was performed to analyse the colony formation efficiency of A549 and A549/DDP cells after indicated treatment. G, The average numbers of colony formed by A549 cells were counted. DDP group vs DMSO group (**P < .01), IL‐7 group vs DDP + IL‐7 group (***P < .001), DDP group vs DDP + IL‐7 group (#*P* < .05). H, The average numbers of colony formed by A549/DDP cells were counted. IL‐7 group vs DMSO group (**P < .01). I, The A549 cells were treated with indicated treatment for 48 h, and the cell apoptosis was measured by flow cytometry. DDP group vs DMSO group (**P < .01), IL‐7 group vs DDP + IL‐7 group (***P < .001), DDP group vs DDP + IL‐7 group (##*P* < .01). J, The A549/DDP cells were treated with indicated treatment for 48 h, and the cell apoptosis was measured by flow cytometry. DMSO group vs IL‐7 group (**P < .01). K, L, The image of A549 and A549/DDP cells apoptosis treated with indicated treatment. M, N, Cell apoptosis was evaluated using HOECHST 33342 assay. Representative images of different groups are showed. Scale bars, 20 µm. Data represent three independent experiments

To determine whether IL‐7 affects the chemotherapeutic sensitivity of NSCLC cells, the effect of IL‐7 alone and of IL‐7 plus cisplatin on A549 cells was determined. As shown in Figure 1A, IL‐7 alone exerted no effects on the cell proliferation, but the combination of IL‐7 and cisplatin significantly decreased the proliferation of A549 cells compared with cisplatin alone treatment. We also observed that IL‐7 decreased the proliferation of A549/DDP cells (Figure 1B). EdU proliferation assays also indicated that the combination of IL‐7 and cisplatin significantly enhanced the sensitivity of A549 to cisplatin compared with cisplatin treatment alone, the percentage of Edu‐positive cells in control group, DMSO group, IL‐7 group, DDP group and DDP + IL‐7 group was 76.81 ± 4.79, 75.39 ± 5.51, 96.96 ± 6.01, 58.96 ± 3.97 and 44.63 ± 2.29, respectively (Figure 1C). The proliferation of A549/DDP cells was decreased by IL‐7 treatment compared with DMSO, the percentage of Edu‐positive cells in control group, DMSO group and IL‐7 group was 70.47 ± 4.15, 71.39 ± 7.30 and 48.29 ± 3.84, respectively (Figure 1D). In addition, colony formation assay showed that the combination of IL‐7 and cisplatin resulted in a decrease in the clonogenic survival of A549 cells compared with cisplatin treatment alone, and the numbers of colony in control group, DMSO group, IL‐7 group, DDP group and DDP + IL‐7 group were 101.33 ± 4.16, 101.00 ± 4.58, 98.00 ± 2.64, 63.67 ± 7.37 and 36.33 ± 4.51, respectively (Figure 1E and G). In A549/DDP cells, IL‐7 treatment alone also decreased the colony formation, and the numbers of colony in control group, DMSO group and IL‐7 group were 80.67 ± 6.03, 80.00 ± 3.61 and 41.33 ± 6.11, respectively (Figure 1F and H). Next, we assessed cell apoptosis of A549 cells under different treatment conditions. As shown in Figure 1I and K, IL‐7 alone exerted no effects on the cell apoptosis, but the combination of IL‐7 and cisplatin significantly increased the cell apoptosis of A549 cells compared with cisplatin alone treatment, and the apoptosis cell rates in control group, DMSO group, IL‐7 group, DDP group and DDP + IL‐7 group were 6.55 ± 0.31, 5.91 ± 0.79, 5.54 ± 0.39, 13.14 ± 1.99 and 31.26 ± 1.88, respectively. IL‐7 treatment alone induced apoptosis of A549/DDP cells, and the apoptosis cell rates in control group, DMSO group and IL‐7 group were 9.94 ± 0.47, 9.85 ± 0.53 and 22.33 ± 1.64, respectively (Figure 1J and L). Similar results were observed in A549 and A549/DDP cells by HOECHST 33342 assays (Figure 1M,N).

### 3.2 IL‐7 enhanced the sensitivity of NSCLC cells to cisplatin by IL‐7R‐JAK3/STAT5 pathway

**Figure 2:**
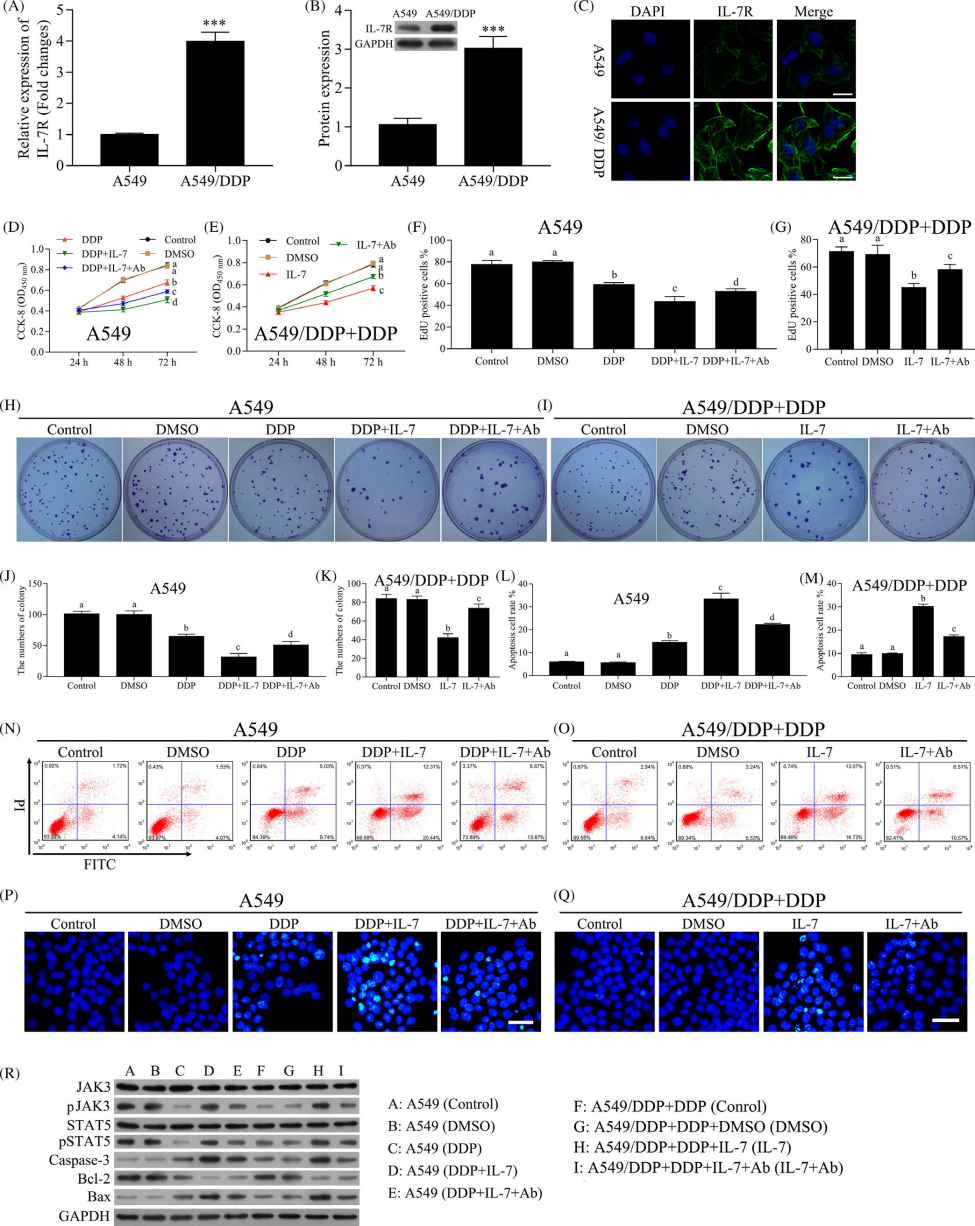
IL‐7 enhanced the sensitivity of NSCLC cells to cisplatin by IL‐7/IL‐7R pathway. A, The mRNA level of IL‐7R in A549/DDP and A549 cells was analysed by qRT‐PCR. B, The levels of IL‐7R in A549/DDP and A549 cells were analysed by Western blot. Densitometry plot of results from the blots is showed. The relative expression levels were normalized to GAPDH. C, Immunofluorescence analysed the protein levels of IL‐7R in A549/DDP and A549 cells. D, E, The A549 and A549/DDP cells were treated with DMSO, cisplatin, IL‐7 or the blocking antibody against the IL‐7 receptor (Ab) alone or combined for 24, 48 or 72 h. The concentration of cisplatin for A549 and A549/DDP cells was 1 and 10 μmol/L, respectively. Cell proliferation analysis using CCK‐8 assay was performed to assess the cell viability after indicated treatment. F, G, The A549 and A549/DDP cells were treated with DMSO, DDP, IL‐7 or the blocking antibody against the IL‐7 receptor alone or combined for 48 h. EdU proliferation assays were performed of A549 and A549/DDP cells after indicated treatment, and the percentage of EdU‐positive cells was quantified. H‐K, Colony‐forming assay was performed to analyse the colony formation efficiency of A549 and A549/DDP cells after indicated treatment. The average numbers of colony were counted. L‐O, The A549 and A549/DDP cells were treated with indicated treatment for 48 h, and the cell apoptosis was measured by flow cytometry. P, Q, Cell apoptosis was evaluated using HOECHST 33342 assay. Representative images of different groups are showed. Scale bars, 20 µm. R, The levels of JAK3, p‐JAK3, STAT5, p‐STAT5, caspase‐3, Bcl‐2 and Bax in A549 and A549/DDP cells after indicated treatment for 48 h were analysed by Western blot. Data represent three independent experiments (average and s.e.m of triplicate samples). The lowercase letters (a, b, c, d) represent statistically significant (*P* < .05). To compare whether there is a significant difference, same letters marked were considered to have no significant difference between the two groups and different letters marked were considered to have significant difference between the two groups

As stated earlier, IL‐7 enhanced the sensitivity of NSCLC cells to cisplatin. We assessed the expression of IL‐7R in A549 and A549/DDP cells. The mRNA level of IL‐7R in A549/DDP cells was higher compared with A549 cells (Figure 2A). The protein level of IL‐7R in A549/DDP cells was higher compared with A549 cells (Figure 2B, C). To investigate the roles of IL‐7/IL‐7R signalling pathway in enhancing the sensitivity of A549 cells to cisplatin, a blocking antibody against the IL‐7 receptor was used. As shown in Figure 2D, E, the administration of IL‐7 combined with cisplatin markedly decreased the proliferation of A549 and A549/DDP cells compared with cisplatin alone treatment. However, the blocking antibody against the IL‐7 receptor reversed the inhibition effects of IL‐7 combined with cisplatin. Similar results were observed with EdU proliferation assays; in A549 cells, the percentage of Edu‐positive cells in control group, DMSO group, DDP group, DDP + IL‐7 group and PPD + IL‐7 + Ab group was 77.64 ± 3.57, 79.81 ± 1.32, 59.15 ± 1.73, 43.36 ± 4.64 and 52.68 ± 2.56, respectively (Figure 2F); and in A549/DDP cells, the percentage of Edu‐positive cells in control group, DMSO group, IL‐7 group and IL‐7 + Ab group was 71.28 ± 3.42, 69.11 ± 6.79, 44.94 ± 3.14 and 58.08 ± 3.83, respectively (Figure 2G). The colony formation efficiency of A549 and A549/DDP cells was significantly inhibited by the administration of IL‐7 combined with cisplatin, and the administration of blocking antibody reversed the inhibition effects (Figure 2H‐K). In A549 cells, the numbers of colony in control group, DMSO group, DDP group, DDP + IL‐7 group and PPD + IL‐7 + Ab group were 101.33 ± 4.04, 100.33 ± 5.50, 65.00 ± 3.61, 31.67 ± 5.69 and 51.00 ± 5.57, respectively (Figure 2H,J). In A549/DDP cells, the numbers of colony in control group, DMSO group, IL‐7 group and IL‐7 + Ab group were 84.00 ± 4.59, 83.00 ± 3.61, 42.15 ± 4.36 and 73.67 ± 4.51, respectively (Figure 2I and K). In addition, the cell apoptosis of A549 cells and A549/DDP cells analysed by flow cytometry was significantly increased by treatment of IL‐7 combined with cisplatin (Figure 2L‐O). The blocking of IL‐7R decreased the numbers of apoptotic cells induced by treatment of IL‐7 combined with cisplatin (Figure 2L‐O). Similar results were observed with by HOECHST 33342 assays (Figure 2P,Q). It has been showed that IL‐7 could activate the tyrosine kinases Jak1 and Jak3 and STAT5 by binding of IL‐7R.^25^, ^26^, ^27^ Western blot assay results showed that the levels ofpJAK3and pSTAT5 in both A549 and A549/DDP cells were decreased by cisplatin treatment but increased by the combined use of IL‐7 and cisplatin, but not affected the expression of JAK3 and STAT5. As expected, the blocking of IL‐7R markedly decreased the levels of p‐JAK3 and p‐STAT5 (Figure 2R). The levels of JAK3 and STAT5 had no significant change under the different treatment conditions (Figure 2R). We also assessed the expression of apoptosis‐related protein. As shown in Figure 2R, the protein levels of caspase‐3 and Bax were increased by cisplatin treatment and increased by the combined use of IL‐7 and cisplatin, and markedly decreased by the blocking of IL‐7R. However, contrasting results were observed for Bcl‐2 protein expression (Figure 2R).

**Figure 3:**
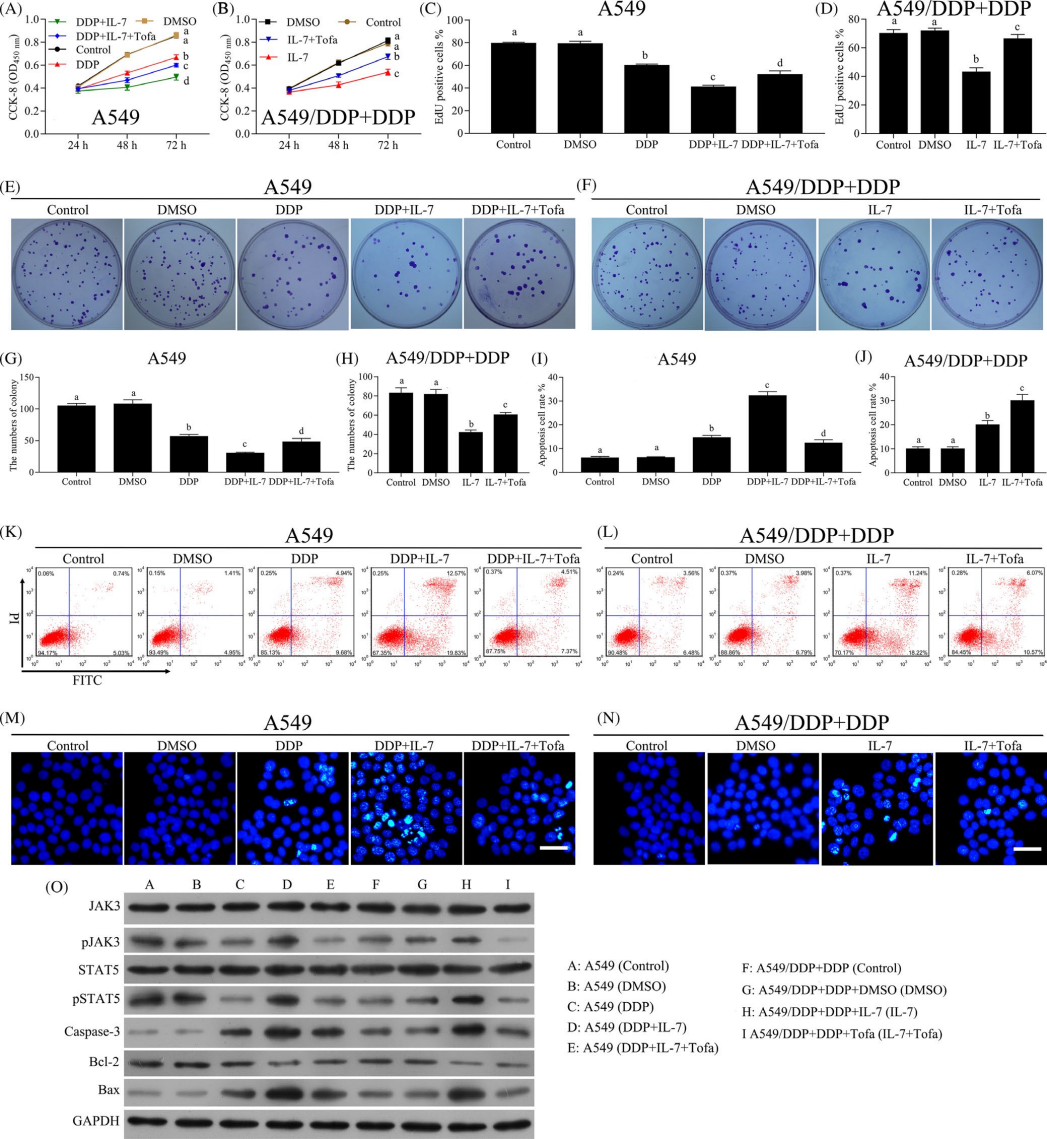
Tofacitinib inhibited the sensitivity of NSCLC cells by IL‐7. The A549 and A549/DDP cells were treated with DMSO, DDP, IL‐7(50 ng/mL) or tofacitinib (pre‐treated, 100 nmol/L) alone or combined as indicated in the figure for 24, 48 or 72 h. The concentration of cisplatin in A549 and A549/DDP cells was 1 and 5 μg/mL, respectively. A, B, Cell proliferation analysis using CCK‐8 assay was performed to assess the cell viability after indicated treatment. C, D, EdU proliferation assays were performed for A549 and A549/DDP cells after indicated treatment for 48 h, and the percentage of EdU‐positive cells was quantified. E‐H, Colony‐forming assay was performed to analyse the colony formation efficiency of A549 and A549/DDP cells after indicated treatment for 48 h. The average numbers of colony were counted. I‐L, The A549 and A549/DDP cells were treated with indicated treatment for 48 h, and the cell apoptosis was measured by flow cytometry. M, N, Cell apoptosis was evaluated using HOECHST 33342 assay. Representative images of different groups are showed. Scale bars, 20 µm. O, The levels of JAK3, p‐JAK3, STAT5, p‐STAT5, caspase‐3, Bcl‐2 and Bax in A549 and A549/DDP cells after indicated treatment for 48 h were analysed by Western blot. Data represent three independent experiments (average and s.e.m of triplicate samples). The lowercase letters (a, b, c, d) represent statistically significant (*P* < .05). To compare whether there is a significant difference, same letters marked were considered to have no significant difference between the two groups and different letters marked were considered to have significant difference between the two groups

To further investigate the involvement of JAK3/STAT5 pathway in enhancing the role of cisplatin sensitivity of NSCLC cells by IL‐7, a novel JAK3 inhibitor was used to inhibit the JAK3 pathway. As shown in Figure 3A, B, tofacitinib reversed the inhibitory effects of IL‐7 combined with cisplatin in the cell proliferation of both A549 and A549/DDP cells. EdU proliferation assays showed a similar result (Figure 3C, D). The colony formation efficiency of A549 and A549/DDP cells was significantly inhibited by the administration of IL‐7 combined with cisplatin, and the administration of tofacitinib reversed the inhibitory effects (Figure 3E‐H). In addition, the cell apoptosis of A549 and A549/DDP cells analysed by flow cytometry was significantly increased by the treatment of IL‐7 combined with cisplatin (Figure 3I‐L). Tofacitinib decreased the numbers of apoptotic cells induced by the treatment of IL‐7 combined with cisplatin (Figure 3I‐L). Similar results were observed with HOECHST 33342 assays (Figure 3M,N). The levels of p‐JAK3 and p‐STAT5 in both A549 and A549/DDP cells were decreased by cisplatin treatment and increased by the combined use of IL‐7 and cisplatin. As expected, the administration of tofacitinib markedly decreased the levels of p‐JAK3 and p‐STAT5 (Figure 3O). The protein levels of caspase‐3 and Bax were increased by cisplatin treatment and increased by the combined use of IL‐7 and cisplatin, and markedly decreased by the administration of tofacitinib (Figure 3O). However, contrasting results were observed for Bcl‐2 protein expression (Figure 3O).

**Figure 4:**
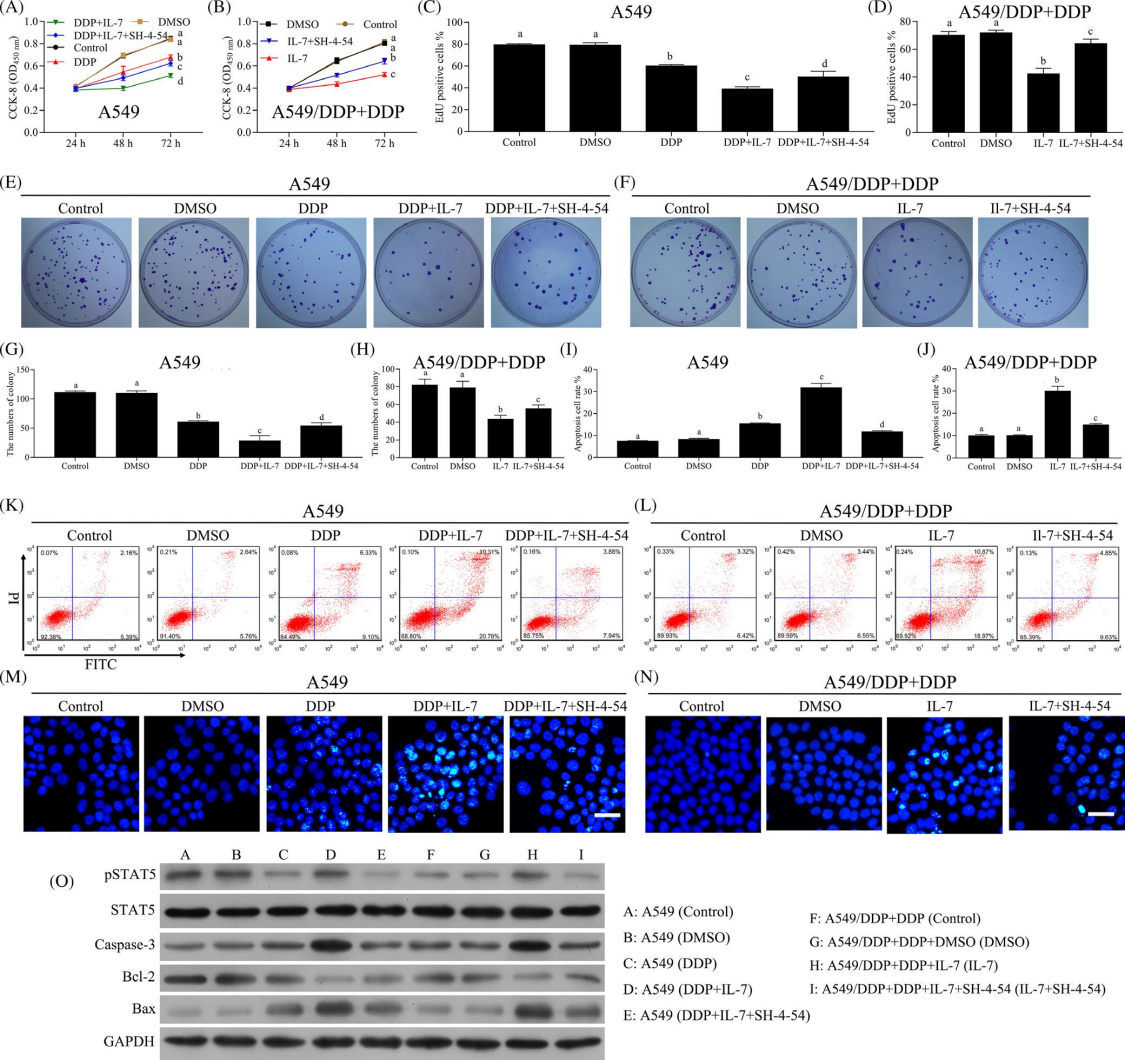
Blocking of STAT5 inhibited the sensitivity of NSCLC cells by IL‐7. The A549 and A549/DDP cells were treated with DMSO, DDP, IL‐7(50 ng/mL) or SH‐4‐54 (pre‐treatment, 25 μmol/L) alone or combined as indicated in the figure for 24, 48 or 72 h. The concentration of cisplatin in A549 and A549/DDP cells was 1 and 5 μg/mL, respectively. A, B, Cell proliferation analysis using CCK‐8 assay was performed to assess the cell viability after indicated treatment. C, D, EdU proliferation assays were performed for A549 and A549/DDP cells after indicated treatment for 48 h, and the percentage of EdU‐positive cells was quantified. E‐H, Colony‐forming assay was performed to analyse the colony formation efficiency of A549 and A549/DDP cells after indicated treatment for 48 h. The average numbers of colony were counted. I‐L, The A549 and A549/DDP cells were treated with indicated treatment for 48 h, and the cell apoptosis was measured by flow cytometry. M, N, Cell apoptosis was evaluated using HOECHST 33342 assay. Scale bars, 20 µm. Representative images of different groups are showed. O, The levels of STAT5, p‐STAT5, caspase‐3, Bcl‐2 and Bax in A549 and A549/DDP cells after indicated treatment for 48 h were analysed by Western blot. Data represent three independent experiments (average and s.e.m of triplicate samples). The lowercase letters (a, b, c, d) represent statistically significant (*P* < .05). To compare whether there is a significant difference, same letters marked were considered to have no significant difference between the two groups and different letters marked were considered to have significant difference between the two groups

The small molecule inhibitor, SH‐4‐54, was used to block STAT5 pathway. SH‐4‐54 pre‐treatment reversed the inhibitory effects of IL‐7 combined with cisplatin in the proliferation of both A549 and A549/DDP cells (Figure 4A, B). Similar results were observed with EdU proliferation assays (Figure 4C, D). For the colony formation efficiency of A549 and A549/DDP cells, the administration of SH‐4‐54 reversed the inhibitory effects of IL‐7 combined with cisplatin (Figure 4E‐H). It was observed that SH‐4‐54 decreased the numbers of apoptotic cells induced by the treatment of IL‐7 combined with cisplatin (Figure 4I‐L). Similar results were observed with HOECHST 33342 assays (Figure 4M,N). The levels of p‐STAT5, caspase‐3 and Bax in both A549 and A549/DDP cells were decreased by SH‐4‐54 under the treatment of IL‐7 combined with cisplatin (Figure 4O). The expression of Bcl‐2 protein in both A549 and A549/DDP cells was increased by SH‐4‐54 with the treatment of IL‐7 combined with cisplatin (Figure 4O). In conclusion, IL‐7 enhanced the sensitivity of NSCLC cells to cisplatin by IL‐7R‐JAK3/STAT5 pathway.

### 3.3 Combination with IL‐7 enhanced the anti‐tumour efficacy of cisplatin in vivo

**Figure 5:**
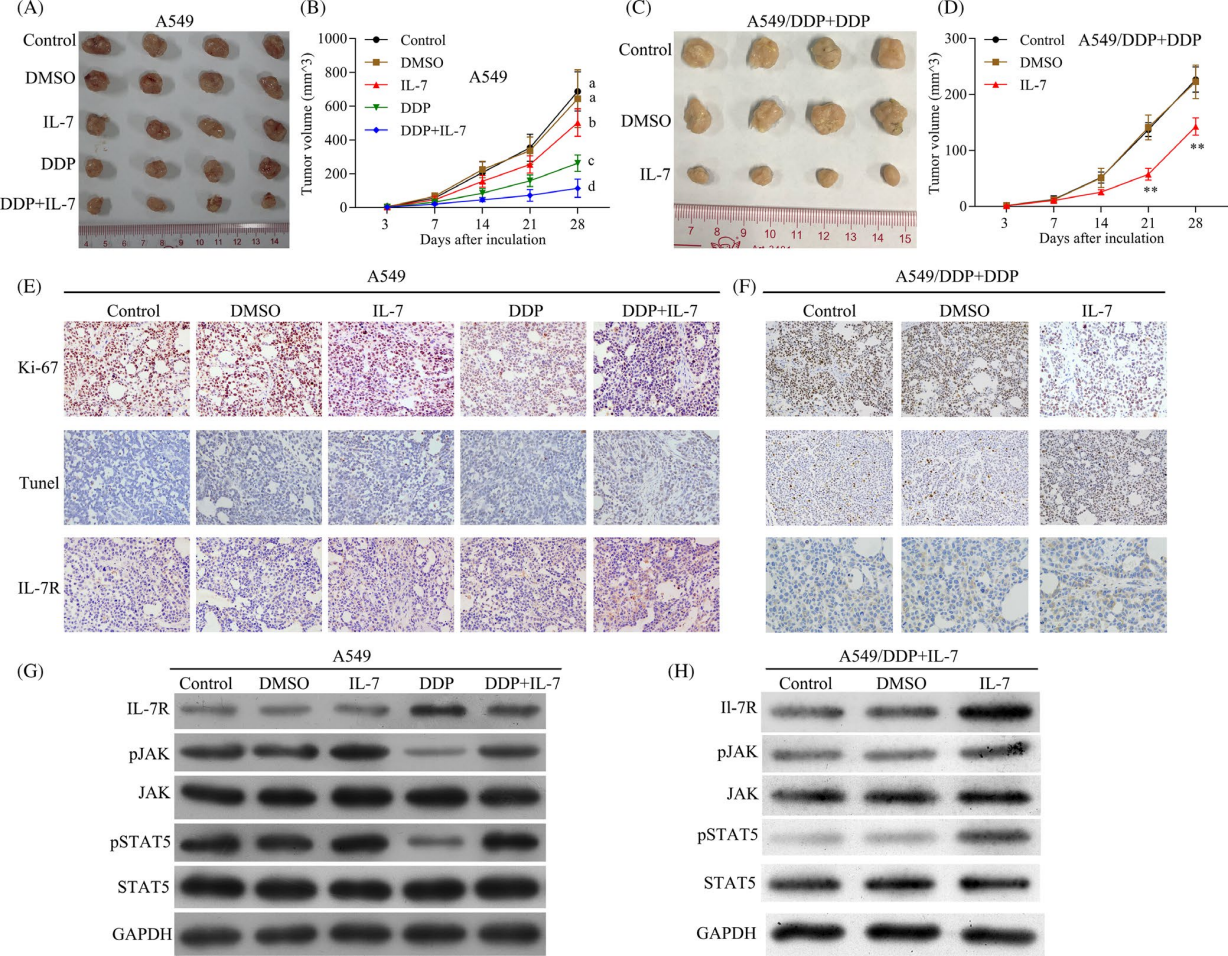
Combination with IL‐7 enhanced the anti‐tumour efficacy of cisplatin in vivo. A549 and A549/DDP cells were used to establish a xenograft model. In A549 mouse tumour model, the mice received the following treatments: DMSO (5%); IL‐7 (5 μg/day); and cisplatin (5 mg/kg) and IL‐7 (5 mg/kg) combined with cisplatin (5 mg/kg) as indicated in the Figure 5A, and in A549/DDP mouse tumour model, the mice were received the following treatments: cisplatin (5 mg/kg); DMSO (5%) combined with cisplatin (5 mg/kg); and IL‐7 (5 μg/day) combined with cisplatin (5 mg/kg) as indicated in Figure 5C. A, B, Representative images of tumours from A549 established tumours after indicated treatment. The changes in tumour volume were monitored and shown (N = 4 per group). C, D, Representative images of tumours from A549/DDP established tumours after indicated treatment. The changes in tumour volume were monitored and shown (N = 4 per group). E, F, Immunohistochemistry analysis of Ki‐67 protein levels in xenograft tumour tissues; TUNEL apoptosis assay analysis of cell apoptosis in tumour tissues. Immunohistochemistry analysis of IL‐7R protein levels in xenograft tumour tissues. Scale bar, 50 μm. G, H, The levels of IL‐7R, JAK3, p‐JAK3, STAT5, p‐STAT5 and GAPDH in tumour tissues were analysed by Western blot. The lowercase letters (a, b, c, d) represent statistically significant (*P* < .05). To compare whether there is a significant difference, same letters marked were considered to have no significant difference between the two groups and different letters marked were considered to have significant difference between the two groups

To investigate whether IL‐7 enhanced the anti‐tumour efficacy of cisplatin in vivo, a xenograft model was established by A549 and A549/DDP cells. The A549 transplanted mice received the following treatments: DMSO; IL‐7; and cisplatin and IL‐7 combined with cisplatin (Figure 5A), and the A549/DDP transplanted mice received the following treatments: control; DDP; and IL‐7 combined with cisplatin (Figure 5C). In A549 cell transplanted model, cisplatin significantly inhibited tumour growth and IL‐7 combined with cisplatin achieved the best therapeutic effect, while the treatment of IL‐7 alone had no effect on tumour growth (Figure 5A and B). In A549/DDP cell transplanted model, IL‐7 combined with cisplatin significantly suppressed the tumour growth compared with cisplatin treatment (Figure C and D). Further, in A549 cell transplanted model, treatment of IL‐7 combined with cisplatin also decreased the expression of Ki‐67 in tumour tissues (Figure 5E) and enhanced cell apoptosis induced by cisplatin in tumour tissues (Figure 5E). Interestingly, the expression of IL‐7R was induced by the treatment of cisplatin, and IL‐7 combined with cisplatin significantly decreased the expression of IL‐7R in tumour tissues (Figure 5E). Western blot assay results showed that p‐JAK3 and p‐STAT5 were markedly increased by IL‐7 under the treatment of cisplatin with on significant effects on the total level of JAK3 and STAT5 (Figure 5G). Moreover, in A549/DDP cell transplanted model, IL‐7 combined with cisplatin significantly reduced the expression of Ki‐67 in tumour tissues compared with cisplatin treatment and enhanced cell apoptosis (Figure 5F). Western blot results showed that p‐JAK3 and p‐STAT5 were markedly increased by IL‐7 under the treatment of cisplatin (Figure 5H). Together, IL‐7 enhanced the anti‐tumour efficacy of cisplatin via JAK3/STAT5 pathway in vivo*.*

## 4 DISCUSSION

Various studies have shown the anti‐tumour activities of IL‐7 by regulating the immune responses against tumour. In lung cancer, IL‐7 enhanced the anti‐tumour response by inhibiting the regulatory T cells.^28^ In murine models of colon cancer and melanoma, the recombinant IL‐7/β‐chain of hepatocyte growth factor HGF hybrid cytokine significantly suppressed the growth of established tumours and the formation of pulmonary metastases.^29^ In a murine model of spontaneous bronchoalveolar cell carcinoma, dendritic cells transduced with an adenovirus vector expressing IL‐7 inhibited the growth of tumour in an organ‐specific manner.^30^ Combination of recombinant IL‐7 and GM‐CSF‐secreting tumour cell immunotherapy markedly enhanced the anti‐tumour efficacy in tumour‐bearing mice.^31^ Mostly, IL‐7 exerts a potentially anti‐tumour effect by enhancing immune responses against tumour IL‐7, which has been showed to have distinct actions on different subsets of T cells. The adjuvant IL‐7 promoted the vaccine‐mediated anti‐tumour immunity with increased IL‐6 production and decreased T helper type 17 cell differentiations and enhanced CD8 T‐cell expansion.^32^

The combination of IL‐7 and IL‐12 synergistically enhances the anti‐tumour immunity by promoting CD3^+^ T‐cell and CD4^+^ T‐cell proliferation.^33^ Also, the combination of IL‐7 and IL‐12 synergistically enhances the anti‐tumour immunity by promoting the proliferation and anti‐tumour function of cytotoxic CD8^+^ T cells.^34^ Most of the previous studies on the anti‐tumour activity were focused on the immune responses. Our study investigated the anti‐tumour role of IL‐7 in tumour cells itself and not the immune responses. Our study showed that IL‐7 enhanced the sensitivity of NSCLC cells to cisplatin via IL‐7R‐JAK3/STAT5 pathway in vitro and in vivo. Sharma S et al reported the anti‐tumour activity of lung tumour‐derived IL‐7 by IL‐7 gene transfer in NSCLC cells.^35^ However, IL‐7 was found to have no effect on tumour growth in mice colon carcinoma and cell resistance to oxaliplatin in CT26 cells.^23^ In glioma cells, IL‐7 has a pro‐tumour role with the enhanced cisplatin resistance and decreased cell apoptosis induced by cisplatin.^36^ The pro‐tumour roles of IL‐7 were also reported in NSCLC for promoting the metastatic process.^37^, ^38^ Our results showed the direct effect of IL‐7 on the A549 cells. However, further studies are required to validate its role in other cancer cells.

IL‐7 binds to the IL‐7R, a heterodimer consisting of IL‐7 receptor α and common γ chain receptor.^39^ IL‐7R is found to be expressed on various cell types, including naive and memory T cells and many others. It has been known to play a critical role in the development of immune cells. In prostate cancer cells, the IL‐7R is found to be upregulated.^40^ In our study, both the mRNA and protein levels of IL‐7R in A549/DDP cells were increased compared with A549 cells. After the IL‐7 binds to IL‐7R, the tyrosine kinases Jak1 and Jak3 are activated, which leads to the activation of STAT5.^25^, ^26^, ^27^, ^41^ The JAK/STAT signalling pathway is a crucial pathway that involves in tumour cell differentiation, migration and proliferation and promotes cell motility by regulating actin dynamics and activating key metastasis‐promoting genes.^42^, ^43^ In particular, STAT3 activation induced by interleukin family of cytokines can promote migration and invasion *via* the regulation of downstream target molecules such as Vimentin, Twist, MMP‐9 and MMP‐7.^44^ Chang Q et al found that the autocrine/paracrine IL‐6/JAK/STAT3 feed‐forward loop has been implicated as a key player of tumour progression and metastasis and an increased level of IL‐6 was found in the invasive breast tumours, with its level positively correlated with advanced stage, confirming a pivotal role of IL‐6 signalling in breast tumour metastasis *in vivo*.^45^ Puja Khanna et al found that GRAM domain‐containing protein 1B (GRAMD1B) was a key signalling molecule that functions to inhibit cell migration in breast cancer by negating JAK/STAT signalling pathway.^46^ In our present study, in both A549 and A549/DDP cells, the levels of p‐JAK3 and p‐STAT5 were decreased by cisplatin treatment and increased by the combined use of IL‐7 and cisplatin. The blocking of IL‐7R markedly decreased the levels of p‐JAK3 and p‐STAT5, with no significant change in JAK3 and STAT5. The inhibition of JAK3 and STAT5 pathway reversed the inhibitory effects of IL‐7 combined with cisplatin and decreased the number of apoptotic cells induced by the treatment of IL‐7 combined with cisplatin. In squamous cell carcinoma of the head and neck, STAT5 activation was involved in the resistance to cisplatin‐mediated apoptosis and inhibiting growth induced by the epidermal growth factor receptor tyrosine kinase inhibitor.^47^ Other studies have reported that miR‐10a silence enhanced the sensitivity of lung cancer to cisplatin via TGFβ/Smad2/STAT3/STAT5 pathway.^48^ In the JAK/STAT signalling pathway, the ligand‐receptor internalization and trafficking to the early endosome were reported to be associated with the signalling intensity,^49^ suggesting that IL‐7/IL‐7R may function to promote ligand‐receptor generate, causing the activation of JAK/STAT signalling. Additionally, C‐terminal truncated STAT5 a/b isoforms, generated by protein processing,^50^, ^51^ retain high‐affinity DNA‐binding activity and present dominant‐negative (DN) character suppressing transcription.^52^, ^53^ Here, whether DN character involved in IL‐7‐mediated IL‐7R‐JAK3/STAT5 signalling pathway contributes to cisplatin‐mediated apoptosis needs to be further investigated.

The efficacy of cisplatin treatment is often limited by intrinsic or acquired resistance to the drug. The possible mechanisms of cisplatin resistance have been elucidated in various cell lines, including increased influx/decreased influx of cisplatin,^54^ increased DNA repair^55^ and activation of signal pathways.^56^ Shen et al found that inhibition of ataxia telangiectasia mutated (AMT) reverses epithelial‐mesenchymal transition (EMT) and decreases metastatic potential of cisplatin‐resistant lung cancer cells through JAK/STAT3/PD‐L1 pathway.^57^ Also, knockdown of miR‐181 reduces autophagy and reduces PTEN/PI3K/Akt/mTOR pathway resulting in cisplatin‐resistant in non‐small‐cell lung cancer.^58^ Moreover, Susan Heavey et al demonstrated that NF‐κB inhibition represents a more promising strategy than PI3K‐mTOR inhibition for treatment in the chemoresistance setting in NSCLC.^59^ In the present study, we only analysed the JAK3/STAT5 pathway involved in IL‐7‐mediated chemotherapeutic sensitivity in non‐small‐cell lung cancer, and whether any other possible mechanisms or cell signalling pathway also contribute to IL‐7‐mediated chemotherapeutic sensitivity in NSCLC needs to be further investigated.

Our results showed that IL‐7 promoted the sensitivity of NSCLC cells to cisplatin via IL‐7R‐JAK3/STAT5 signalling pathway. We highlighted the potential of the combination of IL‐7 and chemotherapy to overcome the cisplatin resistance of NSCLC and delineated the underlying molecular mechanism.

## CONFLICT OF INTEREST

The authors have no conflicts of interest to declare.

## AUTHORS’ CONTRIBUTIONS

LS and ZX contributed equally to this work; LS and BK designed the research study; LS, ZX, QY and YH performed the assays in this research; ZX, YG, FW and BK analysed the data; and LS and BK wrote and revised the manuscript. All authors have read and approved the final manuscript.

## ETHICS APPROVAL AND CONSENT TO PARTICIPATE

Animal experiments were reviewed and approved by the Animal Research Ethics Committee of the Zhujiang Hospital of Southern Medical University.

## Data Availability

The data that support the findings of this study are available from the corresponding author upon reasonable request.
